# Medical Defence Societies

**Published:** 1908-06-27

**Authors:** 


### SPECIAL ARTICLE.

### MEDICAL DEFENCE SOCIETIES.

Probably there is no profession whose members are so much subject to the various forms of speculative and blackmailing legal proceedings as are those who practise medicine. Insincere and unsubstantial as are many of the litigants who annually vex the souls of medical practitioners, their claims are often advocated with great vigour and skill by their legal advisers, and, however flimsy their basis, must always occasion grave anxiety to the respondents. It is well known that a certain amount of mud will stick to the most blameless of the bespattered, and it is by trading on this knowledge that many of those who prosecute medical men hope to be bought off by some form of compromise.

### The Scope and Value of Defence Societies.

To deal with this kind of terror individual action is so helpless that defence societies have sprung into being, and how well they cope with the mischief can be seen by anyone who will trouble himself to read the annual reports, recently issued, of the Medical Defence Union and of the London and Counties Medical Protection Society.

It is not only by defending their members against more or less ill-founded actions for *mala praxis* that these societies benefit their members. Slander, allegations of incompetence or neglect, disputes of any sort arising out of medical practice, and many kindred matters come within their scope; nor are their energies restricted to passive defence, for when occasion demands it the offensive is taken to clear a character or put a stop to an annoyance. Invaluable as are the facilities for protection thus offered, the access to highly experienced and skilled advisers, and the freedom from pecuniary liability in regard to law costs, there are two respects in which defence societies are of even greater service to their members. These are by the relief of worry and anxiety which is brought by the knowledge that the best of assistance is secured to deal with the trouble, and that the possibility of non-success is almost abolished if the case is once taken up on its merits; and by the fact that the vast majority of vexatious and threatened actions collapse at once as soon as it becomes known that their victim has a defence society at his back. It is more the ills they prevent than those they actually cure which form the greatest claim of such associations on the attention of medical practitioners.

### The Two Societies Contrasted.

There are certain points of interest in the reports of both the societies to which we have referred; and these illustrate a few differences between them, though in the main they are so essentially similar. Both societies, we hasten to note, are in a most flourishing condition, numerically and financially. The Defence Union has a membership of 7,477, an increase of 435 over that of 1906; and the

London and Counties of 4,204, an increase of 333. Both have substantial surpluses on the year's working, respectively £700 (nearly) and £520. Both have reserve funds of steadily increasing bulk, and curiously enough in this respect the smaller society just overtops the larger one; but each has accumulated £3,500 from excess revenue in this way. In addition to this there are guarantee funds which increase the resources of the societies to about £10,000 each; the London and Counties may make a levy of ten shillings per member, as well as call up the guarantee of a pound, which is common to the constitutions of both.

So far, then, as finances go there is little difference noticeable; nor is there in the work which is done on behalf of the members. A glance at the reports shows how similar are the difficulties which confront medical men, and how satisfactorily they are met by a defence society. Constitutionally it may be noted that dentists are admitted to the London and Counties Medical Protection Society, whereas, unless they have also a medical qualification, they are excluded from the Defence Union.

### The Question of Indemnity.

But to this minor distinction is, apparently, to be added one much more important. Up to the present neither society has undertaken to indemnify its members against damages awarded against them in the unsuccessful defence of a legal action, nor against the legal costs of the "other side." A proposal to extend the insurance to cover this risk also, has been considered by both Councils; but whereas that of the Defence Union has recommended its rejection, that of the London and Counties is in favour of its adoption.

In discussing the question the Council of the Defence Union conclude that "even if such insurance were possible, it would not be in the interests of either members of the Union or the profession generally, and, indeed, would be against public polity." It is mentioned that legal and expert opinion has been taken on the point, and if the latter part of the pronouncement represents counsel's opinion, it is somewhat surprising that the London and Counties should have been advised in an opposite sense. The Union's solicitor thinks champerty and maintenance might be sustained against the Union if the plan is adopted, though this is disputed by a non-legal correspondent of the *Lancet* (June 13, 1908). On that point the profession must be in the hands of the lawyers, and if they decide in favour of Mr. Hempson's contention the proposed extension of benefits is clearly out of the question.

It is also argued that the mere knowledge of complete indemnity against carelessness might lead to a certain amount of laxity, or might induce courts of law to impute or suspect it. There does not seem to be much in this argument, for most practitioners faced with a legal action are much more worried by the loss of reputation than by the actual case itself. There are, however, certain considerations which deserve to be carefully weighed. For one thing the additional financial responsibility might require an increased subscription. The position of both societies is so strong at present that this increase, even if necessary at all, would be but slight; and the decision whether such protection is worth a slightly higher premium is evidently a matter for the protected. For another, there is a tendency amongst present-day juries to award heavier damages against corporate bodies of presumed affluence than against individuals. Were it known to the jury that the real defendant in an action is a protection society, they might be thereby influenced in the direction of awarding heavier damages than they would do against the practising medical man alone.

In addition, it must not be forgotten that a good deal of extra responsibility must fall on the Council. The societies only undertake to conduct the medico-legal affairs of their members when the respective Councils think fit so to do, or in other words, when the member is not clearly in the wrong. But in the event of either society being cast in heavy damages through the unsuccessful defence of a member, there might be recriminations amongst those members who thought their Council had not exercised a wise discretion in taking up the case. And, similarly, a member whose case the Council should refuse to undertake might complain that he was being deprived of exactly the advantages for which he had subscribed. The addition of insurance against adverse verdicts, damages, and costs, is clearly a matter on which there is a good deal to be said on both sides; and it will be of great interest to the profession to watch the results of the step which the London and Counties Society is taking.

### Minor Differences.

There are a few other respects in which the practice of the societies differs. The Defence Union, for example, spends fifty guineas on accountancy compared with a fifth of that sum debited to the London and Counties. The Union also allows travelling expenses to Council members, and this item, which does not appear on the Protection Society's balance sheet, runs into £166. On the other hand secretarial and clerical salaries are respectively £935 and £882, so that in proportion to revenue, the Union in this respect is more economical. As concerns legal expenses, fluctuations are so marked as to make comparison extremely difficult; but the Protection Society seems to be much less out of pocket proportionally than is the Union, possibly through greater success in recovering awarded costs. Indeed, the balance sheet of the Defence Union makes no mention at all of this saving; presumably the sums mentioned under legal expenses are the net cost of proceedings, and if that is so it is evident that the Union spends a greater percentage of its income in this way than the sister association. That both have obtained the increasing confidence of the profession is proved by their steadily increasing membership; and it is noteworthy that their joint increase is far larger each year than merely that of the number of registered practitioners.

